# Special Issue: “Clinical Diagnosis and Management of Pregnancy Complications”

**DOI:** 10.3390/jcm11195644

**Published:** 2022-09-25

**Authors:** Rinat Gabbay-Benziv

**Affiliations:** 1 Obstetrics and Gynecology Department, Hillel Yaffe Medical Center, Hashalom Street, PB 169, Hadera 38100, Israel; gabbayrinat@gmail.com; Tel.: +972-4-7748224; 2 The Ruth and Bruce Rappaport Faculty of Medicine, Technion-Israel Institute of Technology, Haifa 32000, Israel

Most pregnancies are uneventful and end with a healthy mother and a liveborn baby. Nevertheless, pregnancy poses a risk to the mother, the fetus, and the child. There is no single definition for high-risk pregnancy; however, broadly, any pregnancy in which a pre-pregnancy or new-onset condition poses an actual or potential risk to the wellbeing of the mother or fetus is considered a high-risk pregnancy [1].

In this Special Issue of “Clinical Diagnosis and Management of Pregnancy Complications”, we will address some of the more common complications encountered during pregnancy.

Gestational diabetes mellitus (GDM) is one of the most common medical complication of pregnancy [2,3]. GDM prevalence is increasing and is currently estimated to be between 5 and 25% [3,4] in different populations with different criteria. GDM has serious implications for the mother, the fetus, and the child in the short- and long-term [5]; therefore, it is important to diagnose and treat it. In the short-term, GDM is associated with hypertensive disease during pregnancy, cesarean section, and traumatic deliveries. For the neonate, the main consequences are excessive growth and neonatal hypoglycemia. In the long-term, GDM is associated with increased incidence of type 2 diabetes, as well as metabolic syndrome, for both the mother and the child. Nevertheless, and despite numerous studies in the area, the appropriate diagnosis and management of GDM during pregnancy are still debated. Currently, a diagnosis can be made based on a 75 or 100 g glucose tolerance test, performed either as a diagnostic test for all parturients, according to risk factors (obesity, a family history of diabetes, previous GDM or macrosomia, etc.), or following a positive 50 g glucose challenge test. The accepted thresholds for 100 g OGTT were derived from mathematical extrapolation of values that correlated with the future development of type 2 diabetes in mothers [6]. The values for the 75 g test were determined via selected pregnancy outcomes [7]. Recent studies have shown that using the 75 g glucose tolerance test doubled the prevalence of GDM; however, without any proven clinical perinatal benefit [7,8], controversy remains, and both options are accepted as valid for GDM diagnosis. In this Special Issue, Hanson et al. [9] describe the pregnancy outcomes of a large cohort of un-selected parturients in Estonia over a 7-year period, according to GDM risk factors and diagnoses based on 75 g glucose tolerance test results. In their study, the authors found that the proportion of women with GDM risk factors increased from 43.5% in 2012 to 57.8% in 2018, and the diagnosis of GDM more than doubled (5.2% vs. 13.7%). More importantly, pregnancies in which the mother was predisposed to GDM but had normal 75 g glucose tolerance test results were accompanied by increased odds of delivering a large baby (AOR 2.3 (CI: 1.8–3.0)). As large babies are the hallmark of abnormal glucose exposure during pregnancy, and although 75 g glucose tolerance tests were used in this cohort for GDM diagnosis, this raises concern about the underdiagnosis of GDM using the accepted algorithms. In line with this paradigm, another study in this issue, published by Ramezani Tehrani et al. [10], examined the impact of different GDM diagnostic criteria on the risk of adverse maternal outcomes. In this meta-analysis—which included 49 population-based studies with a total of 1,409,018 pregnant women with GDM and 7,667,546 non-GDM counterparts—the authors found that GDM was associated with an increased risk of adverse maternal outcomes; these included primary cesarean, the induction of labor, maternal hemorrhage, and pregnancy-related-hypertension. Interestingly, this risk was increased regardless of the diagnostic criteria used to diagnose GDM. This finding further stresses the importance of conducting more studies to establish the correct algorithm and diagnostic criteria for GDM diagnosis that will have clinical benefit.

The debate does not end with GDM diagnosis, and controversies continue regarding management and treatment during pregnancy. Currently, establishing good glucose control during pregnancy relies mainly on maternal self-monitoring of blood glucose and fetal weight estimation. In recent years, continuous glucose monitoring (CGM) techniques have become widely available. CGM continuously measures maternal sugar levels, thus offering an alternative to the accepted periodic self-monitoring of blood glucose. Previous data have proven that CGM is safe during pregnancy, and it is now the method of choice for many parturients with pregestational diabetes, mainly with type 1 diabetes, to monitor their blood glucose values. To evaluate CGM’s role and benefits in GDM, Majewska et al. [11] conducted a systematic review that aimed to assess the efficacy of CGM on glycemic control in GDM parturients. In addition, they evaluated the need for pharmacological treatment and perinatal outcomes in GDM parturients with CGM. Fourteen studies were included in their systematic review. The authors concluded that using CGM, when compared to the self-monitoring of blood glucose, improves glycemic control in parturients with GDM. Furthermore, CGM improved qualifications for insulin therapy. CGM demonstrated higher detection of hyperglycemia and hypoglycemia events, especially in insulin-treated parturients. A1c levels were better and gestational weight gain decreased in women using CGM. Nevertheless, neonatal outcomes remained similar without any robust improvement in the rates of macrosomia or neonatal hypoglycemia. Although using CGM to control sugar levels in GDM parturients seems intuitively logical and beneficial, there are still some concerns that should be considered. First, the majority of women with GDM will have good control under a proper diet and physical activity and will have favorable perinatal outcomes, with only mild interruptions to their daily routines. Twenty-four-hour knowledge of their sugar levels might shift their entire focus to their sugar levels, creating stress and anxiety in looking for “perfect” sugar status. This may become problematic, especially as the benefit of CGM has not yet been proven for short- and long-term maternal and child health. Second, as CGM is still considered new technology, there may be selection bias; parturients who are willing to join studies may be more aware and desire better control, thus skewing the results toward optimization of control with CGM. Lastly, the costs of using CGM should be carefully weighed against the yet-to-be-proven theoretical benefits.

Two other common complications of pregnancy are preterm delivery (PTD) and hypertensive disease of pregnancy (HDP). PTD refers to any delivery before 37 gestational weeks. PTD complicates about 5–13% of deliveries worldwide [12] and is the leading cause of neonatal mortality; moreover, it is the most common reason for antenatal hospitalization [13,14,15,16,17]. PTD may be spontaneous, or it may be indicated by a specific maternal or fetal complication. In this issue, a study by Burchard et al. [18] takes us back to the core of establishing PTD using the correct dating methodology. The authors compared the effect of using the last menstrual period (LMP) combined with first-trimester ultrasound-based dating vs. first-trimester ultrasound-based dating alone. The authors demonstrated an improvement in observed biomarker risk predictor performance in parturients who had more certain gestational age dating. In their simulation, a perfect PTD predictor showed a decrease in the AUC of 21% when gestational age was determined using LMP dating and confirmed via ultrasound, and a decrease of about half of that figure when gestational age was determined using ultrasound dating. While ultrasound dating is commonly accepted as a more certain dating method than LMP, its results demonstrate the novel suggestion that confirming LMP using ultrasound does not improve its certainty to the level achieved by using the actual ultrasound dates. This new concept may have wide application in obstetric practices based on accurate gestational age. Important decisions regarding fetal viability, antenatal steroids, the use of neuroprotective magnesium, and even everyday elective labor induction are determined and based on accurate gestation age. Regarding HDP, we present two important studies: The first, published by Yoshizawa et al. [19] explores the etiology and mechanistic base of preeclampsia using gene-expression profile analysis. The second evaluates the variables that are associated with kidney injury in pregnancies that are complicated by preeclampsia with severe features [20].

From a wider perspective on pregnancy complications, age, parity, and interpregnancy interval are crucial variables when predicting adverse outcomes of pregnancy. Over the years, maternal age has increased, particularly in high-income countries [21], and elderly gravida parturients are now relatively common. Numerous data support an increased rate of pregnancy complications with a short interval between pregnancies; however, there are fewer data on long pregnancy intervals, especially when combined with increased maternal age. The dual effect of parity and interpregnancy interval on the risk of pregnancy complications was elucidated in the study by Naeh et al. [22]. The authors performed a population-based retrospective cohort study utilizing all the birth certificate data in the United States in 2017. Parturients who were older than 40 years, and who had a singleton live birth after 24 weeks, were categorized into three groups based on parity and the interval from the last delivery: primiparas, multiparas with a pregnancy interval shorter than 5 years, and multiparas with a pregnancy interval of more than 5 years. The authors found that among multiparas with a pregnancy interval of more than 5 years, adverse outcomes (including PTD of <34 weeks, a birthweight of <2000 g, neonatal seizure, neonatal intensive care unit admission, an Apgar score of <7 at 5 min, or assisted ventilation >6 h) were higher and more like the nulliparity group. Moreover, among parturients older than 40 years, multiparity with a previous pregnancy within 5 years had a significant protective effect against adverse outcomes when compared to nulliparas. This finding has implications when consulting elderly multiparas who have had previous successful pregnancies

As the Guest Editor for this Special Issue, I would like to thank all of the contributing authors for sharing their valuable studies with us. Additionally, I appreciate and thank all the reviewers for their insightful remarks and the JCM team’s support.
